# Parental Awareness, Attitude, and Acceptability of the Human Papillomavirus Vaccine in Jeddah, Saudi Arabia: An Analytical Cross-Sectional Study

**DOI:** 10.7759/cureus.65536

**Published:** 2024-07-27

**Authors:** Rawan A Al Saedi, Manal M Al Daajani, Sami A Alghamdi

**Affiliations:** 1 Preventive Medicine, Ministry of Health, Jeddah, SAU; 2 Communicable Disease Control, Public Health Agency, Ministry of Health, Riyadh, SAU

**Keywords:** vaccine awareness, public health, vaccine hesitancy, hpv-related cancers, parental awareness, hpv vaccine

## Abstract

Background

Human papillomavirus (HPV) is a significant public health concern linked to various cancers and genital warts. Despite the availability of effective vaccines, vaccination rates remain suboptimal in many regions. Understanding parental awareness, attitudes, and acceptability of the HPV vaccine is critical for developing targeted interventions to increase vaccination uptake.

Objective

This study aims to assess parental awareness, attitudes, and acceptability of the HPV vaccine in Jeddah, Saudi Arabia, and identify factors influencing their decision-making regarding HPV vaccination for their children.

Methods

An analytical cross-sectional study was conducted in Jeddah, Saudi Arabia, in 2023. The calculated sample size was 420 parents. Eligible parents with at least one female child aged from nine to 18 years old were invited to participate in the study. A structured questionnaire was administered to parents, collecting data on demographics, knowledge of HPV and its vaccine, attitudes toward vaccination, and reasons for vaccine acceptability or hesitancy. Descriptive statistics were used to summarize the data, and inferential statistics were applied to identify associations between demographic factors and vaccine acceptability.

Results

The study included a diverse sample of parents. Approximately 153 (35.9%) of participants believed the HPV vaccine was effective, while 129 (30.3%) felt the benefits outweighed the risks. Common reasons for vaccinating included protection against sexually transmitted diseases (159, 37.3%) and genital cancers (178, 41.8%). Hesitancy was primarily due to fear of adverse effects (141, 33.1%) and insufficient information (84, 19.7%). Statistical analysis revealed significant correlations between vaccine acceptability and parental education level, employment status, and personal vaccination history.

Conclusion

The findings highlight a moderate level of awareness and acceptability of the HPV vaccine among parents in Jeddah. Educational interventions focusing on vaccine safety and efficacy are essential to address misconceptions and increase vaccination rates. Further research should explore tailored strategies to enhance parental acceptance of HPV vaccination in similar contexts.

## Introduction

The human papillomavirus (HPV), a prevalent virus impacting the genital skin and mucosa, is known to instigate cancers in the oropharynx, vagina, anus, and penis [1]. With over 200 different types, most HPV variants are benign in the general population. However, some variants can lead to genital warts or, more alarmingly, cervical cancer (CC) [1]. A report from the Information Centre on Human Papillomavirus and Cancer and the International Agency for Research on Cancer suggests that CC could be ranked as the third most common gynecological cancer in women in Saudi Arabia. There is no precise data available on the prevalence in Saudi Arabia, but it is known that the HPV genotype is the same as that seen worldwide [2]. According to the Saudi Ministry of Health, the incidence rate of CC is 1.9 per 100,000 women in Saudi Arabia [3]. Notably, in Saudi Arabia, a substantial majority of HPV-positive tumors are infected with HPV-16/18/45 (70% of cases). This infection leads to cancer manifestation approximately five years earlier than the combined HPV-negative and other HPV genotypes [4,5].

CC associated with HPV, particularly high-risk serotypes (HPV-16/18/45), can be effectively prevented in nearly 100% of cases with timely vaccination for females aged 12 to 26 before becoming sexually active [6-10]. The prevalence of CC has notably decreased in most developed countries due to the development and implementation of HPV vaccinations targeting these high-risk serotypes [6,11]. Prior assessments indicate that the majority of Saudi women lack awareness of the availability of HPV vaccination [12]. A study conducted in Saudi Arabia during 2020-2021 revealed a low level of vaccine hesitancy, including for the HPV vaccine. However, most participants (74%) expressed uncertainty about the safety of the new HPV vaccine [13]. In contrast, despite the free availability of the COVID-19 vaccine at various centers in Jeddah, vaccine acceptance remained low [14], indicating a need for further studies to explore the root causes of this discrepancy.

## Materials and methods

Study design and population

The study was an analytical, cross-sectional study conducted in girls' schools in Jeddah, Saudi Arabia, from January to March 2024. The objective was to assess parental awareness, attitudes, and acceptability of the HPV vaccine. The eligible study participants were both men and women aged ≥18, with at least one female child aged between nine and 18 who agreed to participate in the study.

Sampling and sample size

The study utilized a multistage cluster sampling technique to recruit participants from primary healthcare centers (PHCCs) and elementary schools. In the first stage, Jeddah was stratified into seven zones, ensuring all areas were represented. Each zone was inherently comprised of both PHCCs and schools. In the second stage, a random selection of PHCCs and schools was chosen from each zone. Finally, a convenience sampling method was employed to select participants within each chosen PHCC. To reach parents, the researchers contacted them through the school administration for questionnaire completion.

The sample size was estimated based on an expected 50% prevalence of HPV vaccine awareness, attitude, and acceptability among parents in Jeddah. A sample of approximately 385 participants was determined to provide a 95% confidence level with a 5% margin of error. Anticipating potential non-response and considering potential subgroup analyses, the sample size was increased by 10%, bringing the total to 420 participants.

Data collection

A self-administered structured questionnaire, previously validated, was used to collect data [15]. The questionnaire covered various aspects, such as demographic and socioeconomic factors, HPV and vaccine awareness, information sources, perceptions of HPV severity, and factors influencing vaccination decisions. To ensure the questionnaire's validity and make it more accessible to our target population, it was translated from English into Arabic using scientific methods. We did a pilot test, and we can also confirm its validation by expert cultural adoption. Initial reliability tests were conducted for both the knowledge and acceptability scales, with the knowledge total scores revealing a high Cronbach's alpha coefficient of 0.91 and the acceptability scores showing good reliability with a Cronbach's alpha of 0.83. All continuous variables were assessed for normal distribution and found to be skewed.

The data collection was done using an electronic questionnaire that was sent to eligible parents. The form was also shared using a scannable code that directed parents to the online questionnaire. The first part of the questionnaire contained information about the research objectives and target population.

Statistical analysis

Data from the study was entered into an Excel sheet (Microsoft Corporation, Redmond, WA, USA) for data cleaning and coding. The statistical analysis used SPSS Statistics version 28.0 (IBM Corp. Released 2021. IBM SPSS Statistics for Windows, Version 28.0. Armonk, NY: IBM Corp). Descriptive statistics were used to summarize the data, including minimum, maximum, median, and interquartile range (IQR) for continuous variables and frequency and percentages for categorical variables. The primary outcome variable was the total score of HPV vaccine acceptability. Relationships between continuous variables were investigated using Spearman's rank correlation analysis. Significant differences in acceptability total scores between groups were identified using Mann-Whitney and Kruskal-Wallis tests as appropriate. All statistical tests were two-sided, with the significance level set at 0.05. Additionally, linear regression was employed to examine the effect of knowledge on acceptability.

Ethical considerations

This study received approval from Jeddah Health Affairs (approval number: A01815) and was conducted conclusively with the Declaration of Helsinki, the Good Clinical Practice Code, and local regulations. All participants provided informed consent after fully understanding the study's purpose and procedures. Informed consent for all responses was obtained through the electronic form by starting with the explanation of the study and inquiring parents' consent. Utmost care was taken to ensure the confidentiality and anonymity of participant data to maintain privacy.

## Results

In our study, a total of 426 parents participated. The participants were of a median age of 40 with an IQR of 10 years, ranging from 36 to 46 years. Most respondents were mothers (348, 81.7%) and held a bachelor's degree (221, 51.9%). Regarding employment status, 202 (47.4%) were unemployed, while 183 (43%) were full-time employees. Most parents were married (373, 87.6%) and had four or more children (235, 56.1%). The majority of the parents (314, 73.7%) had not vaccinated their children against HPV, and 360 (84.5%) of them had not vaccinated themselves. The participating parents' demographic details and characteristics are displayed in Table 1.

**Table 1:** Parent demographics and analysis of HPV vaccine acceptability * p-value calculated by Kruskal-Wallis test ** significant association using the Kruskal-Wallis test HPV: human papillomavirus

Variable	Groups	Total		Mean rank	p-value*
		N	%		
Relationship with the child	Mother	348	81.7%	214.06	0.495
	Father	46	10.8%	197.46	
	Other	32	7.5%	230.42	
Highest level of education	Less than high school	45	10.6%	191.66	0.214
	Highschool	109	25.6%	199.45	
	Bachelor's degree	221	51.9%	223.71	
	Higher education	51	12%	218.57	
Employment status	Enable to work	17	4.0%	184.35	0.532
	No job	202	47.4%	207.4	
	Student	8	1.9%	257.31	
	Part-time employee	16	3.8%	226	
	Full-time employee	183	43.0%	219.93	
Marital status	Single	8	1.9%	234.44	0.965
	Married	373	87.6%	213.01	
	Divorced	36	8.5%	212.18	
	Widow	9	2.1%	220.56	
How many children do you have?	One	28	6.6%	234.44	0.109
	Two	61	14.3%	213.01	
	Three	98	23.0%	212.18	
	Four or more	239	56.1%	220.56	
Is your child vaccinated against HPV?	Yes	76	17.8%	282.84	<0.001**
	No	314	73.7%	195.73	
	Maybe	36	8.5%	222.14	
Are you vaccinated against HPV?	Yes	34	8.0%	278.19	0.002**
	No	360	84.5%	205.22	
	Maybe	32	7.5%	237.92	

In the current study, the acceptability of the HPV vaccine among parents was evaluated, with scores ranging from a minimum of -8 to a maximum of 9. The median acceptability score was 1, with an IQR from -2 to 4. The relationship with the child, highest level of education, employment status, and marital status did not significantly impact the acceptability of the HPV vaccine (p>0.05). Interestingly, whether the child was vaccinated against HPV (p<0.001) and if the parents themselves were vaccinated against HPV (p=0.002) showed significant effects on vaccine acceptability. For a comprehensive view of the influence of various demographic factors on HPV vaccine acceptability, please refer to Table 1.

The study found that while most parents (285, 66.9%) had heard about the HPV vaccine, their specific knowledge of it varied. For instance, awareness of the vaccine's inclusion in the immunization schedule of their community was low, with 202 (47.4%) being unsure. Additionally, misconceptions existed about who can receive the HPV vaccine, with more parents believing it to be for girls (248, 58.4%) and women (180, 42.4%) than for boys (64, 15.1%) and men (49, 11.5%). This is reflected in the median total knowledge score of 7 (on a scale from -4 to 27), with an IQR of 15 (from 2 to 17), as shown in Table 2.

**Table 2:** Parent's knowledge and attitudes toward HPV vaccination HPV: human papillomavirus, HIV: human immunodeficiency virus, AIDS: acquired immunodeficiency syndrome

Variable	Groups	N	%
Have you ever heard about the HPV vaccine?	Yes	285	66.9%
	No	141	33.1%
Is HPV vaccination included in your autonomous community's immunization calendar?	Yes, only for girls	41	9.6%
	Yes, for boys and girls	30	7.0%
	It is not included	153	35.9%
	Not sure	202	47.4%
In your opinion, who can receive the HPV vaccine? (please indicate all that apply)	Girls	248	58.4%
	Women	180	42.4%
	Boys	64	15.1%
	Men	49	11.5%
	Nobody	41	9.6%
	I am not sure	123	28.9%
HPV vaccine is effective	I strongly agree	71	16.7%
	I agree	126	29.6%
	I disagree	33	7.7%
	I strongly disagree	43	10.1%
	I do not have enough information to answer	153	35.9%
Benefits of the HPV vaccine outweigh the risks	I strongly agree	74	17.4%
	I agree	140	32.9%
	I disagree	40	9.4%
	I strongly disagree	43	10.1%
	I do not have enough information to answer	129	30.3%
In your opinion, the HPV vaccine prevents (indicate all that apply)	Anal cancer	28	6.6%
	Bladder cancer	42	9.9%
	Cervical cancer	110	25.8%
	Oesophagus cancer	6	1.4%
	HIV/AIDS	14	3.3%
	Hepatitis	10	2.3%
	Irritable bowel syndrome	7	1.6%
	Infertility	19	4.5%
	Cancer of the oral cavity	11	2.6%
	Penis cancer	32	7.5%
	Recurrent cystitis	23	5.4%
	Vaginal cancer	120	28.2%
	Vulvar cancer	52	12.2%
	Genital warts	89	20.9%
	None of the above	32	7.5%
	I am not sure	246	57.7%

In the survey of parental opinions on various statements related to vaccination, it was observed that a majority of parents agreed or strongly agreed that vaccination is an effective measure to prevent infectious diseases (310, 72.8%) and that the benefits of vaccination are more significant than the risks (292, 68.5%). However, a considerable proportion of parents (82, 19.3%) agreed or strongly agreed that vaccination is a useless measure. Further details regarding parental perceptions toward HPV vaccination are shown in Table 3.

**Table 3:** Distribution of parental opinions on various statements related to vaccination

Statement	I strongly agree	I agree	I disagree	I strongly disagree	I do not have enough information to answer
Vaccination is an effective measure to prevent infectious diseases	144 (33.8%)	166 (39%)	26 (6.1%)	40 (9.4%)	50 (11.7%)
The benefits of vaccination are more significant than the risks	119 (27.9%)	173 (40.6%)	37 (8.7%)	32 (7.5%)	65 (15.3%)
Vaccination is a useless measure	28 (6.6%)	54 (12.7%)	146 (34.3%)	129 (30.3%)	69 (16.2%)
Parents not vaccinating their children put other people at risk	107 (25.1%)	124 (29.1%)	57 (13.4%)	63 (14.8%)	75 (17.6%)
I am afraid of vaccinating my children	65 (15.3%)	90 (21.1%)	101 (23.7%)	118 (27.7%)	52 (12.2%)

**Figure 1:**
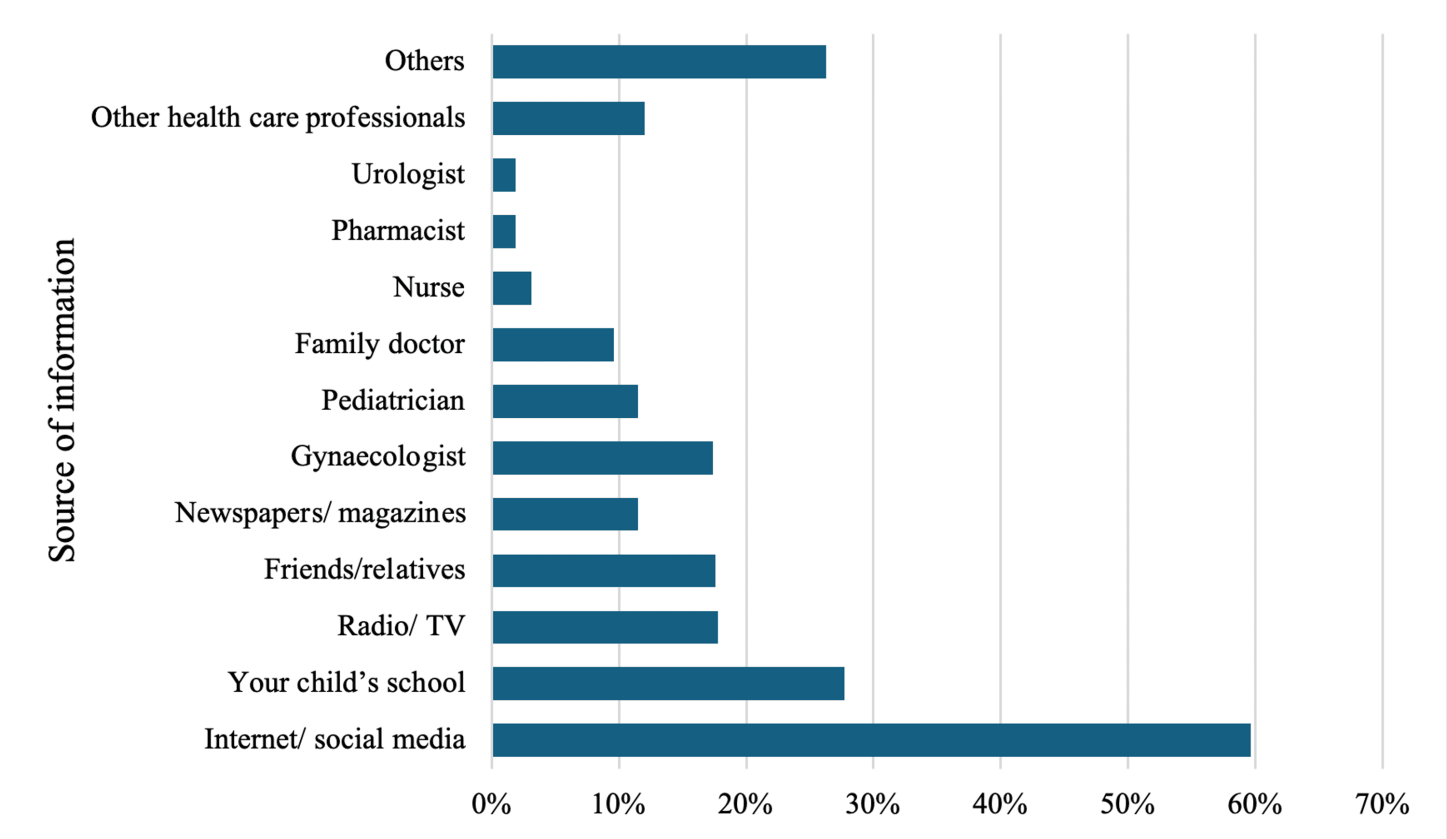
Distribution of respondents' sources of information about HPV vaccination HPV: human papillomavirus, TV: television

The primary sources of information about HPV vaccination, as indicated by the respondents, were the Internet/social media at 59.6%, followed by their child's school at (97, 27.7%). Interestingly, medical professionals were not the primary sources of information, with gynecologists and pediatricians identified by 74 (17.4%) and 49 (11.5%) of the respondents, respectively. Fifty-one (12%) of the respondents mentioned other healthcare professionals, while 112 (26.3%) indicated other unspecified sources for their information about HPV vaccination (Figure 1).

The acceptability of the HPV vaccine among parents did not significantly vary based on the source of information about the vaccination. Parents who received information about the HPV vaccine from the Internet or social media exhibited a mean rank acceptability of 214.5, compared to 212.1 for those who did not utilize these sources, with a p-value of 0.845. Similarly, information from a child's school did not significantly influence vaccine acceptability, with mean ranks of acceptability at 226.8 for those who received information from this source and 208.4 for those who did not (p=0.167). Comparable non-significant findings were observed for other sources of information, including radio/TV, friends/relatives, newspapers/magazines, gynecologists, pediatricians, family doctors, nurses, pharmacists, urologists, other healthcare professionals, and other unspecified sources. Further details can be found in Table 4.

**Table 4:** Comparison of HPV vaccine acceptability based on various sources of information about the vaccine * p-value calculated by Mann-Whitney test HPV: human papillomavirus

Source of information about HPV vaccination	Acceptability mean rank		p-value*
	Yes	No	
Internet/social media	214.5	212.1	0.845
Your child’s school	226.8	208.4	0.167
Radio/TV	230.6	209.8	0.179
Friends/relatives	215.6	213.0	0.868
Newspapers/magazines	236.4	210.5	0.165
Gynaecologist	232.5	209.5	0.142
Pediatrician	237.1	210.4	0.151
Family doctor	234.3	211.3	0.253
Nurse	261.0	212.0	0.156
Pharmacist	251.3	212.8	0.378
Urologist	251.8	212.8	0.373
Other healthcare professionals	239.8	209.9	0.102
Others	223.3	210.0	0.323

**Figure 2:**
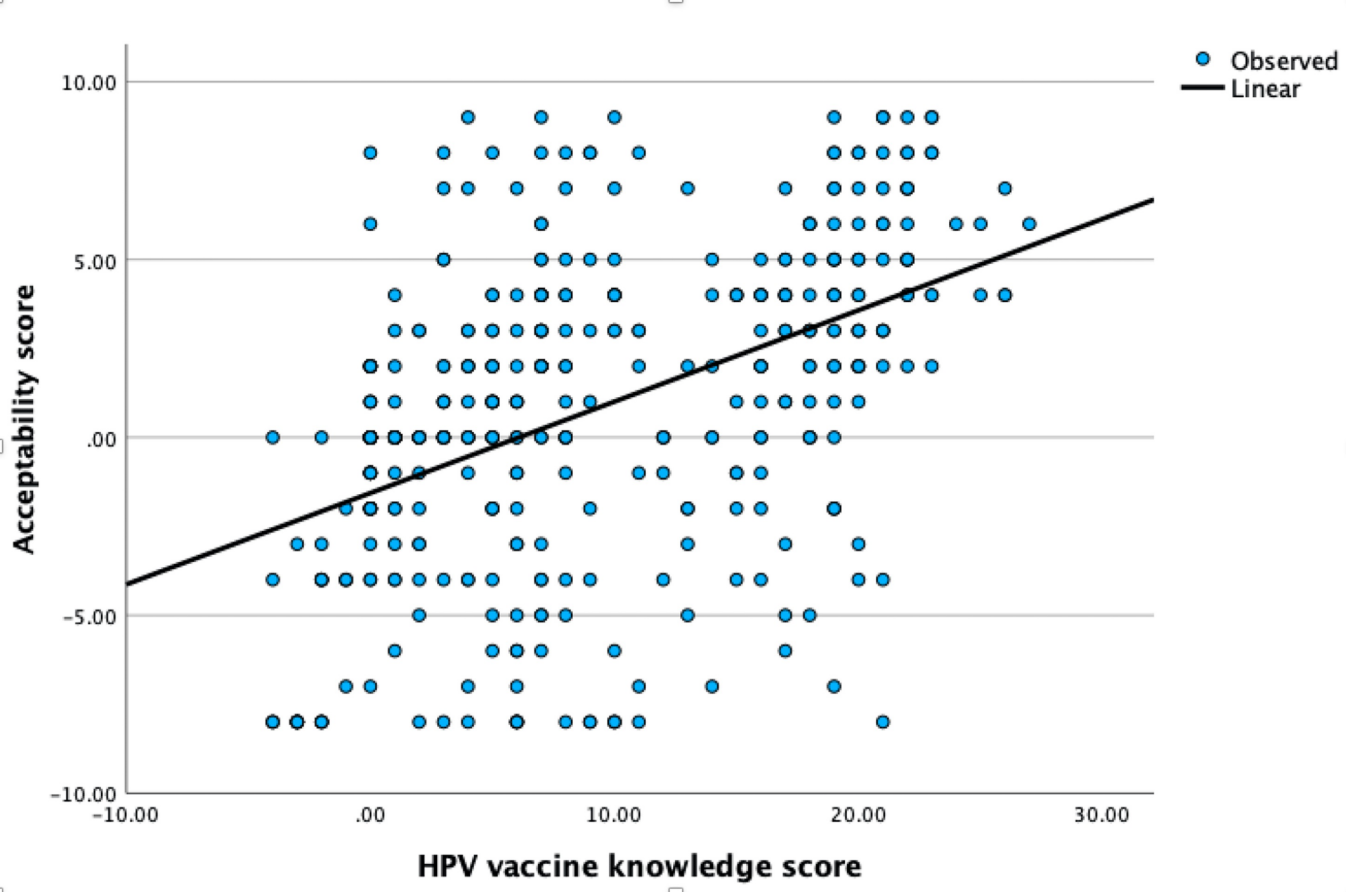
Scatterplot showing the positive correlation between parents' knowledge about the HPV vaccine and its acceptability Spearman's correlation coefficient = 0.514, p<0.001 HPV: human papillomavirus

Our study first conducted a correlation analysis, which revealed a significant positive relationship between the level of knowledge about the HPV vaccine and its acceptability among parents, with a correlation coefficient of 0.514. This suggests a moderately positive relationship, meaning the higher the level of knowledge, the greater the acceptability (p<0.001). A regression analysis was performed based on this significant correlation, giving an R-square value of 0.226. This indicates that nearly 22.6% of the variability in the acceptability scores can be accounted for by the level of knowledge about the HPV vaccine (Figure 2).

## Discussion

This study explored parental awareness, attitudes, and acceptability of the HPV vaccine in Jeddah, Saudi Arabia. Most parents (285, 66.9%) were aware of the vaccine, yet many parents had not heard about it, which is a cause for concern. Our research findings correspond with the previous studies in Saudi Arabia, which have always reported low parental awareness and knowledge of the HPV vaccine [16-18]. These studies also found that a lack of accurate information and misconceptions were the main problems with vaccine acceptance. As an example, Hussain et al. demonstrated that a considerable number (32.3%) of young women or their guardians were not able to acquire basic knowledge about HPV infection and its link to CC [16]. The results of these studies stressed the necessity of special educational projects to solve this vital knowledge problem. Besides, the study also exposed some of the problems and misconceptions, such as behavior, fear, finance, and patriarchy, about the target population for the HPV vaccine [19,20].

A large part of our respondents were under the impression that the vaccine is for girls (249, 58.4%) and women only (181, 42.4%) rather than for boys (64, 15.1%) and men (49, 11.5%). This corresponds to the study conducted by Xie et al. in China, where the absence of information about the advantages of HPV vaccination for males was a problem in promoting uptake [21]. This false notion can result in the loss of the possibility of the complete coverage of families by vaccination, as HPV-related cancers can affect both males and females [22]. Moreover, our research showed that almost half (a combined total of 197 (46.3%)) of the parents agreed with the effectiveness of the HPV vaccine. A sizable percentage of the respondents needed more information on the specific benefits of the vaccination (129, 30.3%), whereas 153 (35.9%) needed more information regarding its effectiveness against HPV-related cancers. This was also noted in a study by Tobaiqy et al. [23], which showed that the main reason for vaccine hesitancy among parents in Saudi Arabia was a lack of understanding about the vaccine's importance. This ignorance about the subject can cause the person to be scared and hesitant about getting the vaccine, thus diminishing the chance of increasing vaccination rates [24].

Wijayanti et al. studied the data from Southeast Asia and found a positive correlation between parental knowledge about the HPV vaccine and vaccine acceptance [25]. Thus, it reaffirms the significance of the approach to the knowledge gaps as the most robust strategy to boost HPV vaccination rates [19,26]. Our study showed that most parents were unaware of the HPV vaccine, but the more information parents had about the vaccine, the more likely they were to accept it. A similar pattern was observed in Indonesia, as found by Sitaresmi et al., where a structured educational intervention increased the parents' understanding and perception of HPV. Thus, this also emphasizes that educational programs help promote HPV vaccination and the possible solution to vaccine hesitancy [27]. Nevertheless, it is necessary to accept regional differences in opinions concerning HPV vaccination. Research conducted in developed countries with well-established HPV vaccination programs usually shows that the population is more aware of and accepts the vaccine, whereas in Saudi Arabia, it is not [6,11]. Therefore, it is clear that cultural and social factors are involved in vaccine hesitancy, and they should be considered when creating interventions [28,29].

The present study has established the Internet/social media as the primary source of information about the HPV vaccine for parents (254, 59.6%). This shows the increasing significance of online platforms in health communication, but at the same time, it also points out the possible inaccuracy or misinformation of the information. Research indicates that social media is a place where there is a lot of false information about vaccines [15]. This shows that credible sources, like health institutions and government websites, should create reliable and easily accessible online resources about HPV vaccination [30]. Schools appeared as the second-most common source of information (27.7%). This research confirms Fauci's findings [31,32], which are supported by the WHO's claim that there are school-based interventions for HPV vaccination awareness and education, which in turn will help to increase the vaccination rate [33]. On the other hand, our research discovered that many parents (172, 40.4%) needed to recognize medical doctors, for example, gynecologists and pediatricians, as the primary sources of information. This, on the other hand, shows that there is a communication gap between healthcare providers and parents on the issue of HPV vaccination.

The main advantage of our study is the use of a reliable questionnaire and a multistage cluster sampling method. This approach made it possible to obtain many parents from Jeddah, Saudi Arabia, who represented the whole population. Nevertheless, the study also has limitations; the fact that the data is gathered through self-reporting may lead to bias. Possible deficiencies, like social desirability bias, resulting from parents overestimating their knowledge or acceptability of the vaccine, can be identified.

## Conclusions

This research highlighted significant gaps in parents' awareness, attitudes, and acceptability of the HPV vaccine in Jeddah, Saudi Arabia. Although the majority of parents were aware of the vaccine, false beliefs regarding the target population and its effectiveness in preventing HPV-related cancers persisted. The fact that misinformation is widespread, primarily through the Internet and social media, shows the need for targeted educational interventions. These strategies fill the existing knowledge gaps, clear the misconceptions, and focus on the fact that the vaccine is effective for both males and females. School-based programs that follow WHO recommendations and improve communication between healthcare providers and parents are the main requirements for the HPV vaccination. By introducing these strategies, we can improve parental decision-making and, thus, see a rise in HPV vaccination rates, which, in turn, will lead to a decrease in HPV-related cancers in Jeddah.
